# Urinary Antiseptics

**Published:** 1915-03-13

**Authors:** 


### RESEARCH AND REMEDIES.

### Urinary Antiseptics.

On a previous occasion the teachings of Mr. J. W. Thomson Walker concerning the use and choice of urinary antiseptics have been quoted. In another paper on this subject, contributed to the *Edinburgh Medical Journal*, he carries the matter a stage further still. Hexamethylene tetramine, commonly known by its proprietary name of urotropin, more nearly approaches the ideal urinary antiseptic than any other yet discovered. It contains in an innocuous form, which passes unchanged through the intestines, circulation, and kidneys, a powerful antiseptic which is liberated in the urine. It should be administered fresh, not combined or mixed with an acid, since the antiseptic power is due to the splitting off of formaldehyde, and this occurs only in acid urine. The only drugs which can be relied on to make an alkaline urine acid are acid sodium phosphate and ammonium benzoate. The latter may be prescribed with hexamethylene tetramine; the former should be given separately. Mr. Walker gives larger doses of hexamethylene than those usually mentioned: fifteen to twenty grains three times a day will result in the urine permanently containing formaldehyde, but if there is great frequency of micturition seven grains every four hours is even better. There is a limit of individual toleration of the drug: symptoms that this limit has been passed are irritability of the bladder, frequent micturition, strangury, and hæmaturia. In predisposed persons even 2½ grains may set up these symptoms, but most people can take twenty without experiencing them. Symptoms of over-dose should be met first of all by withholding the drug and then by giving an alkaline mixture. He prefers hexamethylene tetramine itself to any of the compounds that have been placed upon the market: and regards cystopurin, helmitol, hetraline, and amphotropin as all definitely inferior. He says it is established that no urinary antiseptic claiming to act by the liberation of formaldehyde from hexamethylene tetramine will act in a neutral or alkaline urine. To make the urine acid he gives acid sodium phosphate in twenty-grain doses thrice daily, increasing every other day to thirty, forty, sixty, ninety, 120, or even 150 grains for a dose: the symptom of over-dosage is the appearance of diarrhœa. If ammonium benzoate is used, the dosage is similarly increased, beginning with ten grains and rising to thirty; boric acid may be given with it. It is also important not to provoke a copious diuresis when hexamethylene is being given.

Mr. Walker concludes with five final observations: The dosage of hexamethylene tetramine should be increased until an antiseptic action or the limit of tolerance is reached. The reaction of the urine should be carefully tested, and the acidity, if necessary, raised by means of drugs until the point of dissociation of urotropin is reached. Diuretics and diuretic waters interfere with the splitting of urotropin, and should be avoided during its administration. The Rimini-Burman test for formaldehyde should be in constant use when urotropin is being administered. Urinary antiseptics of the formaldehyde series are harmful or useless, (*a*) in very acute inflammations of the urinary organs, (*b*) in pure tuberculous infections, (*c*) when the urine is alkaline.

